# Depression: [Mental] Health Literacy, Stigma, and Perceived Barriers to Help-Seeking During Transitions Among Undergraduate Nursing Students

**DOI:** 10.3390/nursrep15050172

**Published:** 2025-05-14

**Authors:** Luís Loureiro, Rosa Simões, Amorim Rosa

**Affiliations:** Health Sciences Research Unit: Nursing, Nursing School of Coimbra, 3004-011 Coimbra, Portugal; rosasimoes@esenfc.pt (R.S.); amorim@esenfc.pt (A.R.)

**Keywords:** nursing, mental health literacy, depression, transitions, higher education

## Abstract

**Background/Objectives**: Mental health literacy (MHL) plays a crucial role in transitional processes, influencing, for example, students’ adaptation to higher education and, later, their transition into the workforce. This study assesses MHL about depression among first- and fourth-year nursing students and identifies which MHL components and related variables are associated with a higher likelihood of being in either academic year. **Methods**: A cross-sectional descriptive correlational study was conducted with 478 nursing students (59.4% first-year students; 40.6% fourth-year students), predominantly female (82.2%), with a mean age of 20.41 years (SD = 4.71) and a median age of 19.0 years. **Results**: The results revealed a low level of recognition of depression and knowledge of mental health first aid (MHFA) strategies, including health literacy. Students tended not to hold beliefs based on myths and stereotypes about mental illness and individuals with mental illness and did not have highly stigmatizing views. MHL varied by year of study, with fourth-year students having more adequate MHL levels. **Conclusions**: These findings highlight the need to integrate MHL as a key element of academic success. Health education initiatives that promote mental health, prevention, and MHFA training may serve as effective strategies to enhance MHL among nursing students.

## 1. Introduction

First introduced by Jorm et al. [1] in Australia, mental health literacy (MHL) refers to the beliefs and knowledge about mental disorders (including mental health problems) that enable individuals to recognize, manage, and/or prevent these problems in everyday life [1]. This concept includes five core components: (a) recognition of mental health problems (and mental disorders) to facilitate help-seeking; (b) knowledge of professional help and available treatments; (c) knowledge of effective self-help strategies; (d) knowledge and skills to provide support and first aid to others; (e) knowledge of how to prevent mental illness [2,3].

More recently, the inclusion of other components in the concept, namely positive mental health, has been suggested [4,5] to adopt a salutogenic perspective, focusing interventions on mental health promotion and aligning MHL with health literacy (HL) [6].

More than 25 years after its initial introduction, MHL has assumed a prominent role in the field of public health. It is now recognized both as a social determinant of health and as a foundation for interventions aimed at reducing social stigma and discrimination associated with mental and psychiatric disorders [6].

The MHL of higher education (HE) students, particularly those enrolled in health-related programs such as nursing, has become a growing concern for educational institutions [7,8,9]. This concern is largely driven by evidence of the high prevalence of mental health problems among students [10,11], which may compromise academic success and even threaten their future careers. Common issues include stress, anxiety, depression, and substance abuse, particularly alcohol [12,13]. Notably, some students enter higher education with pre-existing mental health diagnoses, while in other cases, such issues emerge or worsen during their academic journey.

There is limited evidence on the MHL of nursing students, and existing studies suggest modest levels of MHL identical to those of the general population [7,8,9,14,15,16,17,18]. Research has also shown that students often share common beliefs and attitudes toward mental illness that may influence their future work as nurses [8,9]. Investing in MHL is essential, as evidence indicates that individuals with adequate MHL levels are more likely to maintain greater control over their mental state, recognize signs and assess symptoms of psychological distress, and seek help when necessary [2].

Although nursing students face academic challenges and expectations similar to those in other HE programs, the unique nature and characteristics of nursing education place them at greater risk of developing or exacerbating mental health problems. Contributing factors include exposure to human vulnerability and suffering, confrontation with mortality, excessive academic workload, and the continuous need to enhance technical and human skills [19].

Therefore, greater emphasis should be placed on supporting the mental health of nursing students throughout their training, especially at two key moments: the first year, when students enter HE, and the final year, when they graduate and transition to the workforce [20,21,22,23,24].

Among final-year students, factors such as the academic pressures of graduation, uncertainty about their professional future, competitiveness, increased autonomy and decision-making, and responsibility for patient care contribute to the development—or worsening—of mental health problems.

Based on the assumption that MHL increases as students advance in their training and given that final-year students usually have greater theoretical knowledge, more experience in nursing care, reduced social stigma, and enhanced personal and professional skills than first-year students, this study aimed to assess MHL about depression among first- and fourth-year nursing students. Specifically, this study compared the following aspects: (a) the ability to recognize depression in order to facilitate help-seeking and (b) knowledge about the provision of mental health first aid (MHFA).

This study also aimed to evaluate perceived barriers to help-seeking, the intention to seek help, and levels of personal and perceived stigma associated with depression. It also aimed to analyze positive MHL and beliefs about mental illness and individuals with mental illness.

Finally, this study sought to determine whether these variables serve as predictors of students’ year of study. This analysis helps to assess whether fourth-year students have higher levels of MHL compared to first-year students and to explore how this change occurs, including the influence of related variables.

The following steps were undertaken:

- To assess differences in the ability to recognize depression and in the knowledge to provide MHFA, according to the year of study;
- To analyze differences in perceived barriers to help-seeking, intention to seek help, personal and perceived stigma associated with depression, positive MHL and beliefs about mental illness and individuals with mental illness, according to the year of study;
- To identify sociodemographic and MHL-related variables, including familiarity with depression, that are associated with a higher likelihood of being in either the first or fourth year of study.

## 2. Materials and Methods

### 2.1. Research Design

This study used a descriptive, correlational design with a quantitative approach [25]. The STROBE checklist for cross-sectional studies was used to report the findings of this study [26].

### 2.2. Sample and Setting

Data were collected at an HE institution located in the central region of mainland Portugal between September and October 2024. All first- and fourth-year students were invited to participate in the study. All fourth-year students received both theoretical and practical training in psychiatry during the previous year (third year), including clinical placements in mental health institutions.

### 2.3. Data Collection

Data were collected in classroom settings using the Google Forms platform. Researchers were present during each questionnaire administration session. Access to the questionnaire was provided through a QR code. Participants provided electronic informed consent, confirming their understanding of the study’s objectives and their voluntary participation. The average time to complete the questionnaires was 25.17 min.

### 2.4. Instruments

*Sociodemographic questionnaire*: It includes a set of variables such as year of study, age, gender, and parents’ education level.

*Questionnaire for Assessment of Mental Health Literacy*

QuALiSMental [27].

This questionnaire, validated for the Portuguese population, assesses the five components of MHL as defined by Jorm [1]. Psychometric studies conducted in the Portuguese context have demonstrated good reliability and a factor structure consistent with the theoretical components of MHL. The questionnaire is preceded by a clinical vignette describing the case of a 21-year-old woman, Joana, who meets the diagnostic criteria for depression according to the American Psychiatric Association’s Diagnostic and Statistical Manual of Mental Disorders [28]:

*Joana is a 21-year-old woman who has been feeling unusually sad for the last few weeks. She is tired all the time and has trouble falling asleep or staying asleep. She doesn’t feel like eating and has lost weight. She has trouble concentrating on her studies and her grades have sunk. Even day-to-day tasks seem too much for her, so she has put off making decisions. Her parents and friends are very concerned about her.*

This study assessed only two components of the QuALiSMental instrument: (a) recognition of disorders, which includes multiple-choice labels (e.g., depression, *nervous breakdown*, *age crisis*, *psychosis*, etc.); and (b) knowledge and skills to provide support and first aid to others, which includes 10 items rated as “helpful”, “harmful”, or “neither helpful nor harmful” (see Tables 2 and 3 for a list of the labels and the content of the items).

*Personal stigma and perceived stigma scales*.

[29]

The personal stigma and perceived stigma scales [30,31] have been validated for the Portuguese population. However, in the Portuguese version, each scale consists of 7 items rated on a Likert scale from 1 (*strongly disagree*) to 5 (*strongly agree*). The personal stigma items are preceded by the phrase “In my opinion”, while the perceived stigma items use “*Most people believe*”. Both scales use the same items. Each scale is composed of two factors: “weak-not-sick” and “dangerous/unpredictable.”

*Positive Mental Health Literacy Questionnaire*

PosMHLit [32]

This questionnaire consists of 20 items that assess positive MHL and well-being, rated on a Likert scale from 1 (*strongly disagree*) to 5 (*strongly agree*). In line with the original authors’ recommendations, this study used the total score. Studies conducted in the Portuguese context have demonstrated good reliability and construct validity.

*Beliefs Toward Mental Illness Questionnaire*

BtMIq [33]

The BtMIq was developed in the Portuguese context. Its short version consists of 23 items rated on a Likert scale from 1 (*strongly disagree*) to 6 (*strongly agree*). The questionnaire covers the following seven beliefs: belief in dangerousness (BtMIq1), including items such as “People with diagnosed mental illnesses tend to be dangerous.”; belief in inferiority and inability to live in society (BtMIq2), including items such as “People with mental illness have limited ability to live independently because they are unable to take on responsibilities.”; belief in the benefits of patients’ social integration and involvement (BtMIq3), including items such as “People with mental illness are capable of living in their communities if they receive the appropriate support.”; belief in individual responsibility (BtMIq4), including items such as “If people take care of themselves, they can prevent mental illnesses”; belief about psychotropic drugs (BtMIq5), including items such as “The initial treatment of mental illnesses requires the use of medication”; belief in chronicity/incurability (BtMIq6), including items such as “Mental illnesses are cyclical (they return periodically)”; and belief in the efficacy of treatment and rehabilitation (BtMIq7), including items such as “Medication is effective in the improvement of symptoms of mental illness”.

In terms of reliability, Cronbach’s alpha coefficients were as follows: α = 0.81 in the BtMIq1 factor, α = 0.83 in the BtMIq2 factor, α = 0.79 in the BtMIq3 factor, α = 0.71 in the BtMIq4 factor, α = 0.92 in the BtMIq5 factor, α = 0.73 in the BtMIq6 factor, and α = 0.78 in the BtMIq7 factor.

*European Health Literacy Survey*

HLS-EU-PT-Q 16 [34]

The Health Literacy Survey, validated for the Portuguese population, is the short form of the HLS-EU-PT-Q47. It comprises 16 items assessing health literacy across three domains: healthcare, disease prevention, and health promotion. Items are rated on a Likert scale from 1 (*very difficult*) to 4 (*very easy*). In line with the original authors’ recommendations, this study used the total score of the scale [34].

*Barriers to Mental Health Help-Seeking Questionnaire*

*BMHHSQ* [15]

This instrument, used as an annex to QuALiSMental, does not fit specifically into any component but has been applied in various studies [1,35,36]. In this study, its psychometric properties were evaluated through analyses of reliability and construct validity using exploratory factor analysis (EFA). It consists of 9 items rated on a Likert scale from 1 (*completely disagree*) to 5 (*completely agree*) and is preceded by the following instructions: “*Below are different statements about barriers that may stop you from seeking professional help in mental health. If you had a problem like Joana, what would stop you from seeking help?*”

The EFA was performed using the principal components method with Varimax orthogonal rotation, revealing a two-factor solution that explained 54.77% of total variance. The first factor, with five items, is called *Personal barriers to seeking help* and explains 33.26% of the variance. This factor includes items such as “Concern that the person might feel negatively about me” and “concern about what other people might think”. The second factor is called *Barriers to accessing specialized professional help* and includes items such as “Concern that I might have difficulties getting an appointment”. It explains 21.52% of the total variance. In terms of reliability, the value of internal consistency using Cronbach’s alpha coefficient was α = 0.83 for the first factor and α = 0.71 for the second factor.

### 2.5. Data Analysis

Data were analyzed using SPSS, Version 29.

Appropriate summary statistics were calculated, as well as absolute and percentage frequencies where appropriate based on variable type. The normality of the distribution was assessed using the Lilliefors-corrected Kolmogorov–Smirnov test. Homogeneity of variance was assessed using Levene’s test.

To address the study’s main objectives, the following tests were used:

- Two-proportion *z*-test, with Cohen’s *h* as the measure of effect size [37], where *h* = 0.20: small effect size; *h* = 0.50: medium effect size; and *h* = 0.80: large effect size.
- Chi-square test for RxC contingency tables using Cramer’s *V* as the effect size with *Df* = 2, where *CV* = 0.07: small effect size; *CV* = 0.21: medium effect size; and *CV* = 0.35: large effect size [37].
- Student’s *t*-test for independent samples, with Cohen’s *d* as the effect size, where *d* = 0.20: small effect size; *d* = 0.50: medium effect size; and *d* = 0.80: large effect size [38].
- Binary logistic regression (BLR) by blocks using the forward method. Validation of the BRL assumptions was carried out by analyzing the residuals and diagnosing influential cases [39]. Prior to analysis, the nominal qualitative variables were dichotomized (coded as “0” and “1”). The criterion or predictor variable was coded as “0” for the first-year group and as “1” for the fourth-year group. LRA analysis was selected due to its suitability for binary outcome variables, allowing for the classification of students into either the first or fourth year. This model enabled the identification of key predictor variables influencing the likelihood of a nursing student belonging to a specific academic year.

### 2.6. Ethical Considerations

This study was conducted in accordance with the principles outlined in the Declaration of Helsinki for research involving human subjects. It was approved by the Ethics Committee of the Health Sciences Research Unit: Nursing of the Nursing School of Coimbra, Portugal. All participants remained anonymous, and data were stored in a secure, encrypted online archive accessible only to the study’s authors. The survey included an electronic informed consent form for students to sign. The consent form also included permission for the publication of the research data.

## 3. Results

The sample consisted of 478 nursing students, of whom 284 (59.4%) were enrolled in the first year and 194 (40.6%) in the fourth year. Regarding gender, 82 (17.2%) were male and 396 (82.8%) were female. The mean age was 20.41 years (SD = 4.71), with a median age of 19.0 years.

In terms of familiarity with mental health, 315 students (66.0%) reported having a family member or close friend in a situation similar to that described in the vignette, while 163 students (34.0%) did not. Additionally, 242 students (50.7%) had sought or received treatment for mental health problems. A total of 115 students (24.11%) had a friend in a similar situation and had sought professional help themselves.

Concerning the first objective, “to assess the differences in the ability to recognize depression, as well as in the knowledge to provide first aid to others, according to the year of study”, Table 1 indicates significant differences in the use of the following labels: *age crisis* (z = 5.186; *p* < 0.001; h = 0.52), *nervous breakdown* (z = 4.160; *p* < 0.001; h = 0.39), and *anxiety* (z = 2.416; *p* < 0.05; h = 0.22). In all three cases, these labels were cited more frequently by first-year students. All effect sizes (h) were in the medium range.

Another relevant finding is the low rate of correct identification of the vignette as a case of depression: 12.0% of first-year students correctly identified the case as depression, rising to 27.3% among fourth-year students. The differences were statistically significant, with a medium effect size (*z* = −4.252; *p* < 0.001; *h* = 0.39).

A large proportion of students mentioned the labels *nervous breakdown* (78.8% in the first year and 61.3% in the fourth year) and *anorexia* (23.7% in the first year and 18.6% in the fourth year).

Regarding *knowledge to provide first aid to others, according to the year of study*, the chi-square test results (Table 2) revealed statistically significant differences in the following items: “Talk to her firmly about getting her act together” [MHFA_i2] (χ^2^_(2)_ = 71.350; *p* < 0.001; *CV* = 0.39), “Ask her whether she is feeling suicidal” [MHFA_i5] (χ^2^_(2)_ = 14.963; *p* < 0.001; *CV* = 0.18), “Not acknowledge her problem, ignoring her while she gets over it” [MHFA_i8] (χ^2^_(2)_ = 6.284; *p* < 0.05; *CV* = 0.11), “Keep her busy to keep her mind off problems” [MHFA_i9] (χ^2^_(2)_ = 35.924; *p* < 0.001; *CV* = 0.28), and “Encourage her to become more physically active” [MHFA_i10] (χ^2^_(2)_ = 16.806; *p* < 0.001; *CV* = 0.18).

In all items with statistically significant differences, more adequate knowledge was associated with fourth-year students. Effect sizes ranged from 0.18 to 0.28, indicating medium effects.

Regarding the objective “To analyze the differences in the perceived barriers to mental health help-seeking, the intention to seek help, the personal and perceived stigma associated with depression, positive MHL, and beliefs about mental illness and people with mental illness, according to the year of study”, several t-tests for independent samples were performed. As shown in Table 3, statistically significant differences were found in the following beliefs: dangerousness (t = 2.996; *p* < 0.05; d = 0.28), inferiority and inability to live in society (t = 7.449; *p* < 0.001; d = 0.69), social integration and involvement (t= −4.263; *p* < 0.001; d = 0.40), efficacy of psychotropic drugs (t = −3.262; *p* < 0.001; d = 0.30), chronicity/incurability (t = 2.828; *p* < 0.01; d = 0.26), and efficacy of treatment and rehabilitation (t = −3.291; *p* < 0.05; d = 0.31). Effect sizes ranged from d = 0.26 to d = 0.69, corresponding to small (d = 0.26; d = 0.28; d = 0.30; d = 0.31; and d = 0.40) and medium (d = 0.69) effect sizes.

The mean scores for adjusted and positive beliefs, such as the belief in *social integration and involvement* and the *efficacy of psychotropic drugs*, were higher among fourth-year students. In contrast, the mean scores for negative beliefs were higher among first-year students.

Regarding personal stigma, statistically significant differences were found in both subscales: *weak-not-sick* (*t* = 4.576; *p* < 0.001; *d* = 0.43) and *dangerous/unpredictable* (*t* = 2.633; *p* < 0.01; *d* = 0.25). For perceived stigma, statistically significant differences were found only in the *weak-not-sick* subscale (*t* = −2.750; *p* < 0.01; *d* = 0.26).

Regarding barriers to mental health help-seeking associated with depression, statistically significant differences were found in two dimensions: *personal barriers to seeking help* (*t* = −3.058; *p* < 0.01; *d* = 0.29) and *barriers to seeking professional help* (*t* = −3.449; *p* < 0.001; *d* = 0.29). In both cases, effect sizes were small.

Statistically significant differences were also found in the last two variables: *positive mental health* (*t* = −4.362; *p* < 0.001; *d* = 0.28) and *health literacy* (*t* = −2.948; *p* < 0.01; *d* = 0.41), with higher mean scores among fourth-year students.

The HL variable was categorized according to the authors’ proposal [34]. Therefore, 19.1% of students had an inadequate level of HL, 29.8% had a problematic level, and 51.2% had an adequate level. As regards the level of HL by year of study, a statistically significant association was found (χ^2^_(2)_ = 6.613; *p* = 0.037; *CV* = 0.12), which shows a tendency for most first-year students to have inadequate HL levels, while most fourth-year students were more likely to have adequate HL levels.

Finally, to identify which sociodemographic variables and MHL-related variables (including familiarity with depression) predict students’ year of study (first or fourth year), a binary LRA was conducted using a blockwise approach.

Table 4 shows that the predictors involved in the model are, related to the recognition of disorders, the *age crisis* (*b =* −1.18; *p* < 0.001; *OR* = 0.31) and *nervous breakdown* (*b =* −0.57; *p* < 0.05; *OR* = 0.31) labels, as well as the following items related to knowledge and skills about MHFA: *talk to her firmly about getting her act together*—MHFA_i2 (*b =* −3.31; *p* < 0.001; *OR* = 0.04), *ask her whether she is feeling suicidal*—MHFA_i5 (*b =* 0.71; *p* < 0.01; *OR* = 2.02), *keep her busy to keep her mind off problems—*MHFA_i9 (*b =* −0.79; *p* < 0.001; *OR* = 0.45), and *encourage her to become more physically active*—MHFA_i10 (*b =* 1.37; *p* < 0.001; *OR* = 3.94).

Regarding the beliefs, the predictors were the belief in inferiority and inability to live in society—BtMIq2 (b = −1.23; *p* < 0.001; OR = 0.29), belief in social integration and involvement—BtMIq3 (b = 0.56; *p* < 0.05; OR = 1.74), belief in efficacy of psychotropic drugs—BtMIq5 (b = 0.47; *p* < 0.01; OR = 1.60), and belief in efficacy of treatment and rehabilitation (b = 0.47; *p* < 0.05; OR = 1.59). The last variable to enter the model was the weak-not-sick subscale of the perceived stigma scale (b = 0.35; *p* < 0.01; OR = 1.42).

Model fit was assessed using pseudo-R-square measures: the Cox–Snell and Nagelkerke R-square. The scores indicated good model fit: R^2^_Cox-Snell_ = 0.33 and R^2^_Nagelkerke_ = 0.45. The area under the curve (AUC) was 0.86, indicating very good discrimination and a strong model.

## 4. Discussion

The following discussion considers that the nursing students who participated in this study reported a high level of familiarity with mental illness, particularly through family members and/or close friends with such diagnoses. Half of the sample reported having sought or received treatment for mental health problems. Although traditionally viewed as a facilitator of MHL, this familiarity may paradoxically hinder it—either due to negative experiences with mental health services or professionals or as a result of excessive normalization stemming from close exposure to individuals with mental illness (family/friends). This normalization can lead to the trivialization of symptoms and reduce the capacity to recognize problems and disorders.

As a component of MHL, the ability to recognize a mental health problem or disorder is a prerequisite for seeking help [1,2,3]. The findings in this study are consistent with previous research [7,8,9,40], particularly regarding the frequency of labels considered appropriate. While the prevalence of the *depression* label was over 80.0% in both academic years, the most frequently chosen labels were *stress*, *anxiety*, and *psychological, mental, and emotional problems*.

A careful reading of the vignette indicates that the symptoms described are consistent with a diagnosis of depression according to the DSM-5 criteria [28]. Although depression may co-occur with anxiety, they are distinct clinical conditions.

Two labels generally considered inappropriate—*age crisis* and *nervous breakdown*—showed significant differences between first- and fourth-year students, with the *age crisis* label being more frequent (*h* > 0.50). *Anorexia* was also a frequent label.

The *nervous breakdown* label was more frequent among first-year students (approximately 4/5 of the sample), but it is still used by more than half (>60.0%) of fourth-year students. This non-specific label is commonly used in everyday language to describe any change in mental health. In this context, these labels hinder accurate identification.

While the percentage of students who correctly identified depression increased from 12.0% in the first year to 27.3% in the fourth, this result can be interpreted in two ways. On the one hand, the overall rate of accurate identification of depression remains modest (18.3%), consistent with findings from previous studies [41,42,43]. On the other hand, progression through the nursing program appears to improve MHL, albeit to a lesser extent than desirable [44,45].

Regarding the component of knowledge on how to provide first aid and support to others, the findings are consistent with those reported in previous studies [41,42,43]. Active listening was favored and perceived by nearly all participants as useful in the process of helping others, which is aligned with the guidelines of the MHFA program [46,47]. It is also encouraging that a substantial majority of students considered it helpful to *suggest she seek professional help and make an appointment for her to see a GP with her knowledge*. Similar findings have been reported in other studies [7,8,9,40,41,42,43,47].

Among other positive or help-seeking interventions, *Ask her whether she is feeling suicidal* stands out. This result has also been reported in several studies conducted across diverse contexts [7,8,9,40,46]. Slightly more than one-third of participants perceived this strategy as helpful. This finding is concerning, as many individuals—including nursing students, students from other academic programs, and even health professionals—consider this action to be harmful or fail to recognize its importance. This hesitation is commonly rooted in the misconception that directly asking someone about suicidal thoughts may trigger or encourage such behavior [40].

For strategies such as *talk to her firmly about getting her act together*, *not acknowledge her problem, ignoring her*, and *keep her busy to keep her mind off problems*, the results indicate a substantial effect of training. The findings suggest that students’ academic progression can improve MHL, particularly in challenging myths and stereotypes associated with mental illness. This finding is consistent with previous research [46].

Concerning beliefs about mental illness, individuals affected by it, and even the associated social stigma, most studies indicate that students tend to initially endorse attitudes that foster stigma and social distancing [48,49].

In the present study, however, the scores related to beliefs, as well as personal and perceived stigma, do not suggest that nursing students hold particularly negative or stigmatizing views of mental illness. These findings are partly consistent with previous research [50,51,52,53].

Mean scores reflect consistency with positive beliefs and inconsistency with negative ones. As shown in previous studies [52,53], students’ progression through the nursing program seems to reduce negative beliefs and promote positive ones. This effect was especially significant for the *belief in inferiority and inability to live in society* (effect size = 0.69).

Participation in the program contributed to greater endorsement of positive beliefs about mental illness (belief in social integration and involvement, belief in efficacy of psychotropic drugs, belief in efficacy of treatment and rehabilitation), while also reducing negative beliefs (belief in dangerousness and belief in chronicity/incurability). These changes suggest that evidence-based training within nursing education helps challenge common myths and stereotypes. This shift is supported by academic exposure to mental health and psychiatric content, including clinical placements, which progressively encourage students to reconsider pre-existing beliefs.

In addition to the negative beliefs previously discussed, systematic literature reviews [48,49] have shown that young people often demonstrate limited knowledge of mental health issues, hold negative beliefs, and have high levels of personal stigma. They do not see depression as an illness but rather as a sign of personal weakness and say that people suffering from depression can even be dangerous to themselves and others.

In the present study, both personal and perceived stigma scores were lower among fourth-year students than first-year students, which suggests that attending the program can reduce social stigma, a finding that aligns with previous research [54,55].

Regarding perceived barriers to help-seeking, research shows that a significant number of young people are reluctant to seek mental health help [1,2,3,56]. The most frequently cited reason is personal stigma [57], followed by limited access to mental health services and professionals. Nursing students are no exception [58], and the results of this study are consistent with the evidence [57,58,59]. Participants reported higher scores for personal barriers to help-seeking than for access to professional help. Fourth-year students demonstrated a reduced influence of personal factors, while first-year students were more affected by access-related personal factors.

Research on positive mental health literacy (MHL) among nursing students remains limited, largely because this approach is relatively new compared to traditional MHL approaches stemming from Jorm’s work [6]. Nonetheless, the findings from this study indicate overall satisfactory results, with high average scores—particularly among fourth-year students—indicating good knowledge of MHL in both academic years.

HL is also recognized as an important variable for nursing students, particularly in relation to how HE institutions incorporate HL into their curricula [60]. Existing literature suggests that between 35.0% and 45.0% of nursing students have inadequate or even problematic levels of HL [61,62,63,64]. In this study, these figures were around 50% of the sample, which is a worrying result for nursing students.

Finally, the findings from the model suggest that the nursing program has a mitigating effect on factors associated with students’ low MHL. Specifically, being a fourth-year student is associated with a 69% lower likelihood of selecting the *age crisis* label and a 44.0% lower likelihood of reporting the *nervous breakdown* label when compared to first-year students.

With regard to MHFA strategies, fourth-year students were 96% less likely to find the strategy *talk to her firmly about getting her act together* helpful and 55.0% less likely to find the strategy *keep her busy to keep her mind off problems* helpful when compared to first-year students.

Fourth-year students were 102.0% more likely to find the strategy *ask her whether she is feeling suicidal* helpful and 294.0% more likely to find the strategy *encourage her to become more physically active* helpful compared to first-year students.

In terms of beliefs, the greater the student’s *belief in inferiority and inability of patients to live in society*, the less likely they were to be in the fourth year. For each one-point increase in the *belief in social integration and involvement of patients*, the likelihood of being a fourth-year student increased by 74%; for each one-point increase in the *belief in efficacy of psychotropic drugs*, the likelihood of being a fourth-year student increased by 60.0%; for each one-point increase in the *belief in efficacy of treatment and rehabilitation*, the probability of the student being in the fourth year increased by 60.0%; and, finally, for each one-point increase in *perceived stigma*, specifically in the *weak-not-sick* dimension, the likelihood of being a fourth-year student increased by 42.0%.

These findings support the idea that, as students progress through the program, positive beliefs become stronger and more balanced, while negative beliefs become less prevalent. The data also suggest that the program plays a key role in reducing negative beliefs and in increasing students’ knowledge of MHFA. Additionally, students demonstrate improved recognition of symptoms and a greater appreciation of their importance, which is particularly evident in the decline in the use of inappropriate labels that often perpetuate stigma surrounding mental illness.

It is important to note that the fourth-year students underwent theoretical training in psychiatry and mental health, as well as clinical training in psychiatry, during the previous academic year (third year), a factor that may contribute to the interpretation of these results.

## 5. Conclusions

The results of this study offer a valuable contribution to HE institutions focused on nursing education. They highlight the importance of investing in MHL from the onset of academic training, as this promotes MH, well-being, and academic success in its multiple dimensions.

In line with the conceptual definition of MHL, which encompasses both knowledge and beliefs about mental disorders, this study reveals two key findings. First, there is no indication of a strong social stigma among students regarding mental illness, which is encouraging for future healthcare professionals whose daily work will focus on human relationships. Second, there is a limited ability to recognize depression, potentially reflecting a devaluation of the symptoms. This is particularly concerning given that half of the students have inadequate or problematic MHL levels.

Although participants had some knowledge of MHFA strategies, a significant proportion reported being unfamiliar with them. This finding highlights the need to implement MHFA programs that reinforce specific components where knowledge gaps exist. The results also underscore the importance of integrating MHL into nursing curricula.

Future research should assess MHL across all academic years and explore other variables, such as students’ MH status and well-being.

## Figures and Tables

**Table 1:** Absolute and percentage distribution of the labels used to identify the problem described in the vignette, according to the year of study (N = 478).

Labels	Year		z ^(a)^	ES ^(b)^
	First Year (*N* = 284)	Fourth Year (*N* = 194)		
Age crisis	84 (29.7)	19 (9.8)	5.186 ***	0.52
Mental illness	162 (57.2)	124 (63.9)	−1.461 ^ns^	0.14
Bulimia	23 (8.2)	14 (7.2)	0.365 ^ns^	0.03
Stress	266 (94.0)	178 (91.8)	0.947 ^ns^	0.09
Nervous breakdown	221 (78.8)	119 (61.3)	4.160 ***	0.39
Alcohol abuse	7 (2.5)	6 (3.1)	−0.408 ^ns^	0.04
Psychosis	15 (5.3)	8 (4.1)	0.589 ^ns^	0.06
Psychological problems	255 (90.1)	175 (90.2)	−0.036 ^ns^	0.01
Anorexia	67 (23.7)	36 (18.6)	1.334 ^ns^	0.13
Anxiety	271 (95.8)	175 (90.2)	2.416 *	0.22
Depression	234 (82.7)	162 (83.5)	−0.234 ^ns^	0.02
Schizophrenia	5 (1.8)	2 (1.0)	0.657 ^ns^	0.07
Correct identification	34 (12.0)	53 (27.3)	−4.252 ***	0.39

^(a)^ two proportion *z*-test; ^(b)^ effect size *h* measure; * *p*< 0.05; *** *p*< 0.001; ns: non-significant

**Table 2:** Absolute and percentage distribution of students’ opinions on strategies for providing MHFA, according to the year of study (N = 478).

Knowledge About MHFA	Year	Helpful	Harmful	NHH ^(a)^	χ^2^	ES
1. Listen to her problems in an understanding way [MHFA_i1]	1st 4th	277 (58.7) 195 (41.3)	1 (33.3) 2 (66.7)	3 (100.0) --- (0.0)	2.563 ^(1)^ (ns)	0.08
*Total % in row*		98.7	0.6	0.6		
2. Talk to her firmly about getting her act together [MHFA_i2]	1st 4th	52 (96.3) ^(b)^ 2 (3.7)	116 (43.1) 153 (56.9) ^(b)^	113 (72.9) ^(a)^ 42 (27.1)	71.350 ***	0.39
*Total % in row*		11.5	56.4	32.3		
3. Suggest she seek professional help [MHFA_i3]	1st 4th	253 (57.6) 186 (42.4)	5 (62.5) 3 (37.5)	23 (74.2) 8 (25.8)	3.334 ^(1)^ (ns)	0.08
*Total % in row*		91.8	1.7	6.5		
4. Make an appointment for her to see a GP with her knowledge [MHFA_i4]	1st 4th	203 (57.7) 149 (42.3)	23 (74.2) 8 (25.8)	55 (57.9) 40 (42.1)	3.249 (ns)	0.08
*Total % in row*		73.4	6.5	20.1		
5. Ask her whether she is feeling suicidal [MHFA_i5]	1st 4th	82 (47.7) 90 (52.3) ^(b)^	105 (68.2) ^(b)^ 49 (31.8)	94 (61.8) 58 (38.2)	14.963 ***	0.18
*Total % in row*		35.4	33.2	33.2		
6. Suggest she have a few drinks to forget her troubles [MHFA_i6]	1st 4th	5 (71.4) 2 (28.6)	263 (58.1) 190 (41.9)	13 (72.2) 5 (27.8)	1.762 (ns)	0.06
*Total % in row*		1.5	94.8	3.8		
7. Rally friends to cheer her up [MHFA_i7]	1st 4th	177 (59.8) 119 (40.2)	21 (63.6) 12 (36.4)	83 (55.7) 66 (44.3)	1.029 (ns)	0.05
*Total % in row*		62.1	6.9	31.0		
8. Not acknowledge her problem, ignoring her [MHFA_i8]	1st 4th	3 (100.0) --- (0.0)	264 (57.6) 194 (42.4) ^(b)^	14 (82.4) ^(b)^ 3 (17.6)	6.284 **	0.11
*Total % in row*		0.6	95.8	3.6		
9. Keep her busy to keep her mind off problems [MHFA_i9]	1st 4th	133 (71.1) ^(b)^ 54 (28.9)	53 (38.4) 85 (61.6) ^(b)^	95 (62.1) 58 (37.9)	35.924 ***	0.28
*Total % in row*		39.2	28.7	32.1		
10. Encourage her to become more physically active [MHFA_i10]	1st 4th	207 (54.3) 174 (62.5) ^(b)^	5 (62.5) 3 (37.5)	69 (77.5) ^(b)^ 20 (22.5)	16.806 ^(1)^ ***	0.18
*Total % in row*		79.7	1.7	18.7		

^(a)^ NHH = neither helpful nor harmful; ^(b)^ adjusted residuals > 1.96; ES: effect size; ^(1)^ based on the Fisher–Freeman–Halton Exact Test; ns: non-significant; ** *p* < 0.01; *** *p* < 0.001. ns: non-significant.

**Table 3:** Beliefs about mental illness and people with mental illness, personal and perceived stigma, barriers to seeking help, health literacy, and positive mental health literacy by year of study (N = 478).

	Year	Mean	SD	t	ES
***Beliefs about mental illness and people with mental illness***					
Dangerousness (BtMIq1)	1st	2.17	0.78	2.996 *	0.28
	4th	1.96	0.73		
Inferiority and inability to live in society (BtMIq2)	1st	2.66	0.55	7.449 ***	0.69
	4th	2.29	0.52		
Social integration and involvement (BtMIq3)	1st	4.09	0.52	−4.263 ***	0.40
	4th	4.30	0.53		
Individual responsibility (BtMIq4)	1st	2.32	0.65	0.274 ^ns^	0.03
	4th	2.30	0.54		
Efficacy of psychotropic drugs (BtMIq5)	1st	2.44	0.74	−3.262 **	0.30
	4th	2.66	0.65		
Chronicity/incurability (BtMIq6)	1st	2.82	0.60	2.828 **	0.26
	4th	2.67	0.60		
Efficacy of treatment and rehabilitation (BtMIq7)	1st	3.61	0.63	−3.291 *	0.31
	4th	3.80	0.55		
***Personal stigma***					
Weak-not-sick	1st	1.82	0.60	4.576 ***	0.43
	4th	1.59	0.47		
Dangerous/unpredictable	1st	1.63	0.52	2.633 **	0.25
	4th	1.51	0.51		
***Perceived stigma***					
Weak-not-sick	1st 4th	2.89 3.11	0.88 0.89	−2.750 **	0.26
Dangerous/unpredictable	1st 4th	2.48 2.53	0.74 0.78	−0.670	0.06
***Barriers to mental health help-seeking***					
Personal barriers to seeking help (PBHSS-1)	1st	3.28	0.96	3.058 **	0.29
	4th	3.00	0.99		
Barriers to accessing specialized professional help (PBHSS-2)	1st	2.52	0.80	−3.449 ***	0.32
	4th	2.78	0.86		
***Other variables***					
Positive mental health literacy (PosMHLit)	1st	84.28	8.90	−4.362 ***	0.28
	4th	87.82	8.47		
Health literacy (HLS-EU-PT-Q)	1st	11.38	4.13	−2.948 **	0.41
	4th	12.45	3.58		

ns: non-significant; * *p* < 0.05; ** *p* < 0.01; *** *p* < 0.001.

**Table 4:** Logit coefficients of the binary logistic regression model of the variable: year of study according to the sociodemographic variables, variables related to MHL, beliefs, stigma, and HL (N = 478).

Variables	B	SE	Wald	df	*p*	*OR*	Lower	Upper
Age crisis	−1.18	0.32	13.872	1	<0.001	0.31	0.17	0.57
Nervous breakdown	−0.57	0.26	4.993	1	<0.05	0.56	0.34	0.93
MHFA_i2 (helpful)	−3.31	0.80	17.069	1	<0.001	0.04	0.01	0.18
MHFA_i5 (helpful)	0.71	0.25	7.916	1	<0.01	2.02	1.24	3.31
MHFA_i9 (helpful)	−0.79	0.25	10.224	1	<0.001	0.45	0.28	0.74
MHFA_i10 (helpful)	1.37	0.31	19.592	1	<0.001	3.94	2.15	7.23
BtMIq2	−1.23	0.24	26.123	1	<0.001	0.29	0.18	0.47
BtMIq3	0.56	0.24	5.235	1	<0.05	1.74	1.08	2.80
BtMIq5	0.47	0.17	7.406	1	<0.01	1.60	1.14	2.24
BtMIq7	0.47	0.21	5.181	1	<0.05	1.60	1.07	2.41
Perc. stigma (weak-not-sick)	0.35	0.14	6.512	1	<0.01	1.42	1.09	1.86
Constant	−3.94	1.40	7.945	1	0.005	0.019		

## Data Availability

Data supporting the findings of this study are available from the corresponding author upon request.

## Public Involvement Statement

There was no public involvement in any aspect of this research.

## Guidelines and Standards Statement

This quantitative study used the STROBE checklist to report the findings of cross-sectional studies in epidemiology.

## Abbreviations

The following abbreviations are used in this manuscript:

BLR	binary logistic regression analysis
DSM	Diagnostic and Statistical Manual of Mental Disorders
HE	higher education
HL	health literacy
MH	mental health
MHFA	mental health first aid
MHL	mental health literacy
